# Severity of Influenza A(H1N1) Illness and Emergence of D225G Variant, 2013–14 Influenza Season, Florida, USA

**DOI:** 10.3201/eid2104.141375

**Published:** 2015-04

**Authors:** Nicole M. Iovine, J. Glenn Morris, Kristianna Fredenburg, Kenneth Rand, Hassan Alnuaimat, Gloria Lipori, Joseph Brew, John A. Lednicky

**Affiliations:** University of Florida, Gainesville, Florida, USA (N.M. Iovine, J.G. Morris Jr., K. Fredenburg, K. Rand, H. Alnuaimat, G. Lipori, J.A. Lednicky);; Florida Department of Health, Gainesville (J. Brew)

**Keywords:** influenza virus, H1N1 subtype, pandemic, hemagglutinin, influenza, acute respiratory distress syndrome, respiratory tract disease, sialic acid, viruses, Florida, D225G polymorphism, genetic changes

## Abstract

Despite a regional decline in influenza A(H1N1)pdm09 virus infections during 2013–14, cases at a Florida hospital were more severe than those during 2009–10. Examined strains had a hemagglutinin polymorphism associated with enhanced binding to lower respiratory tract receptors. Genetic changes in this virus must be monitored to predict the effect of future pandemic viruses.

In 2009, a novel influenza virus, influenza A(H1N1)pdm09, emerged. The resulting pandemic disproportionately affected persons <65 years of age (*1*), but illness caused by the virus was similar in severity to that caused by seasonal influenza (*2*). As the 2013–14 influenza season progressed, physicians at a Florida hospital noted that patients <65 years of age were affected in numbers similar to those seen in 2009–10, but with increased severity. To investigate these observations, we obtained the number of influenza admissions during the 2013–14 season, characterized pathologic findings in deceased patients, sequenced subtype H1N1 viruses, and assessed receptor-specific characteristics.

## The Study

**Figure 1:**
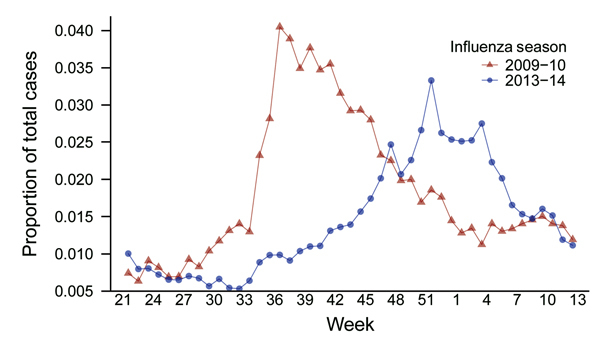
Proportion of all emergency department visits attributable to influenza-like illness, 2009–10 versus 2013–14 influenza seasons, Florida Department of Health Region 3, Florida, USA. Emergency department visits for influenza-like illness are shown as a proportion of total emergency department visits. Week 21 corresponds to the end of May for both influenza seasons, and week 12 corresponds to the end of March.

Studies were approved by the University of Florida Institutional Review Board and the Florida Department of Health. Data on influenza-like illness (ILI) were obtained from the 13-county Florida Department of Health Region 3 (2010 population 2.2 million) of Florida’s Electronic Surveillance System for the Early Notification of Community-based Epidemics (*3*). ILI-associated emergency department visits were defined as those having a chief complaint containing the words influenza or flu or the word fever plus cough or sore throat. During August 2009–March 2010, a total of 12,022 (2.18%) of 549,101 illnesses among all patients visiting a hospital emergency department met the ILI case definition (Figure 1). During the same period in 2013–14, a total of 12,496 (1.67%) of 746,560 illnesses met the case definition (p<0.0001 by χ^2^ test) (Figure 1).

During September 1, 2013–March 21, 2014, we tracked test results for influenza-positive inpatients and outpatients at a major tertiary referral center for the north Florida region. Rapid point-of-care and respiratory virus panel tests were performed: 808 (>97%) of 826 positive samples were influenza A virus, and of 181 samples subtyped, 163 (90%) were H1N1 virus. During this period, 387 patients with laboratory-confirmed influenza were admitted to the hospital; 15 died, yielding an influenza-associated death rate of 3.9%. Each deceased patient had >1 risk factors for severe influenza, placing them in a high-risk group as defined by the Centers for Disease Control and Prevention (*4*) (Table). Documentation of influenza vaccination was found for only 3 of the 15 deceased patients. Similar data for the 2009–10 influenza season were not available.

**Table:** Characteristics of 15 patients who died from influenza virus infection, Florida, USA, 2013–14*

Characteristic	No. (%)
Sex	
M	8 (53.3)
F	7 (46.7)
Age, y	
18-29	1 (6.7)
30-49	7 (46.6)
50-65	3 (20.0)
>65	4 (26.7)
Influenza type	
A	4 (26.7)
H1N1	11 (73.3)
BMI	
30-40	5 (13.3)
>40	2 (20.0)
Co-occurring conditions	
Asthma/COPD	5 (33.3)
Diabetes	3 (20.0)
Heart disease	3 (20.0)
Immunosuppression	5 (33.3)
Liver disease	1 (6.7)
Neurologic disorder	1 (6.7)
Renal disease	4 (26.7)
Smoking	2 (13.3)
Influenza vaccination record	3 (20.0)

*COPD, chronic obstructive pulmonary disease.

To identify patients admitted to the hospital’s medical intensive care unit (MICU) for influenza during August 1, 2013–March 31, 2014, we searched the University of Florida’s Health Integrated Data Repository for patients with diagnosis codes from the International Classification of Diseases, 9th Revision (ICD-9), for influenza (487) or novel influenza (488) listed among the first 10 diagnoses. We used the number of patients admitted with these codes as a marker of disease severity. The mean age of these patients was 50.2 years, which did not differ greatly from the mean age of 43.4 years in 2009–10 (Mann-Whitney U test). Of 1,024 patients admitted to the MICU in 2013–14, a total of 49 had influenza, yielding an admission rate of 4.8%. During the same period in 2009–10, a total of 10 of 821 MICU patients were admitted with influenza, yielding an admission rate of 1.2%. This difference was statistically significant (p<0.0001 by 2-tailed Fisher exact test). The risk ratio for MICU admission for influenza in 2013–14 versus 2009–10 was 3.7 (95% CI 1.9–7.3). Therefore, although the overall number of ILIs in the community was lower in 2013–14 versus 2009–10 (Figure 1), illness in 2013–14 was more severe, as reflected by a higher MICU admission rate.

**Figure 2:**
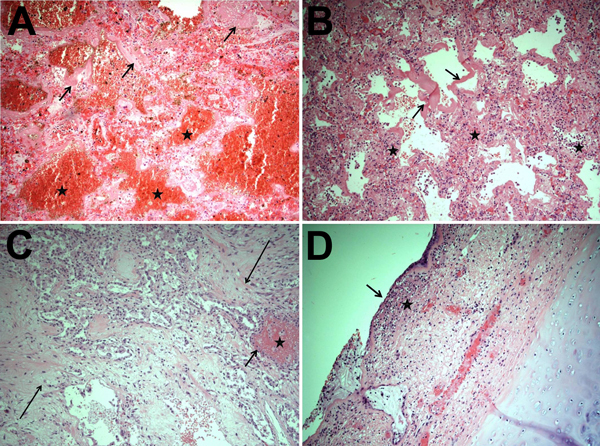
Results of autopsies for patients who died from influenza A(H1N1) virus infection during the 2013–14 influenza season in Florida Department of Health Region 3, Florida, USA. A) Marked intraalveolar hemorrhage (stars) and hyaline membranes (arrows) were seen during the early phase of diffuse alveolar damage and were the most common findings in the 5 autopsy cases reviewed. B) Hyaline membranes, a proteinaceous exudate replacing the alveolar walls (arrows), are prominent in this lung section, as is interstitial edema. Also, a lymphocytic infiltrating inflammation, characteristic of viral pneumonia, is shown (stars). These histologic findings correspond clinically to changes that occur during acute respiratory distress syndrome, and they occur 3–7 days after lung injury. C) The alveolar parenchyma has been replaced predominately by spindled, proliferative fibroblasts (long arrows) and hyperplastic type II pneumocytes (short arrow), indicative of the organizing phase of diffuse alveolar damage seen ≈1 week or more after lung injury. A small focus of intraalveolar hemorrhage is also present (star). D) Necrotizing tracheitis, characterized by desquamation of the tracheal columnar epithelium (arrow) and submucosal acute inflammation (star), is shown. Original magnification ×100 for all panels.

Of the 15 deaths in 2013–14, twelve occurred in the MICU, all among the 49 patients admitted for influenza (death rate 24.5%). The mean age of deceased patients was 48.2 (range 25–89) years; 40% were <40 years of age. Acute respiratory distress syndrome and influenza A virus infection were diagnosed in all 12; none had bacterial infection before testing positive for influenza. Ten of the 12 viruses were subtyped by using the respiratory virus panel: all 10 were H1N1 virus. We reviewed autopsy findings for 5 of the H1N1 virus–infected patients. Although most influenza viruses infect trachea and upper respiratory tract cells (*5*), histopathologic examination of respiratory samples from all 5 patients revealed marked intraalveolar hemorrhage and diffuse alveolar damage (Figure 2, panels A, D), indicating lower respiratory tract disease that clinically manifested as acute respiratory distress syndrome. In 4 patients, neutrophilic alveolar infiltrates consistent with pneumonia were seen (Figure 2, panel B). Changes consistent with the chronic stage of diffuse alveolar damage were noted in 2 patients (Figure 2, panel C). Two others showed necrotizing tracheitis (Figure 2, panel D); swab samples from these tissues were positive for H1N1 virus.

Hemagglutinin of human influenza viruses typically binds to cells bearing sialylglycan receptors configured in an α2-6 orientation. However, some A(H1N1)pdm09 virus isolates also bind to α2-3 sialylglycan receptors (*6*). In humans, α2-6 sialylglycans are found throughout the respiratory tract, but α2-3-sialylglycans are mainly expressed on lower respiratory tract cells (*7*). The permissiveness of wild-type MDCK cells and those overexpressing α2-6-sialylglycan receptors (MDCK-SIAT2,6-UF) and α2-3-sialylglycan receptors (MDCK-SIAT2,3-UF) was assessed by monitoring cytopathic effects of virus strains derived from patients (*8*,*9*). Cytopathic effects were first apparent and were extensive in MDCK-SIAT2,6-UF cells, followed by MDCK-SIAT2,3-UF, then wild-type MDCK cells. These results suggest that the H1N1 virus isolates in our study use both α2-6- and α2-3-sialylglycan receptors, providing a potential mechanism by which lower respiratory tract disease can occur.

The molecular basis for the ability of the H1N1 viruses to cause severe lower respiratory tract disease was first noted with the 1918 pandemic H1N1 virus (*10*). A single amino acid change of aspartic acid at position 225 to glycine (D225G) enabled binding to α2-3- and α2-6-sialylglycans (*10*,*11*). The association of this polymorphism with severe and lower respiratory tract disease was also noted with the A(H1N1)pdm09 virus from small subsets of patients during the 2009–10 influenza season (*12*–*14*). To determine if this polymorphism existed in the H1N1 viruses isolated in our study, we sequenced viral RNA corresponding to the coding regions of all 8 influenza virus genomic segments from viruses isolated from or detected in 7 patient samples (GenBank sequences KJ645758–KJ645765 and KJ645774–KJ645791) (*8*). The consensus sequences were similar to those of key American A(H1N1)pdm09 virus isolates, and like a subset of those isolates, our 7 H1N1 isolates harbored the D225G polymorphism.

None of the 7 isolates harbored the H275Y neuraminidase polymorphism that confers oseltamivir resistance (*15*), and in vitro test results confirmed oseltamivir susceptibility (data not shown). To determine whether polymorphisms in the 3′ and 5′ untranslated regions were associated with the 2013 H1N1 virus, we sequenced 1 isolate by using rapid amplification of cDNA ends (FirstChoice RLM-RACE; Ambion, Bleiswijk, the Netherlands). No substantial differences were detected between this virus and the original A(H1N1)pdm09 virus or circulating contemporary H1N1 viruses.

Our work was subject to certain limitations. First, the use of ICD-9 codes for diagnostic information is imprecise. To mitigate this, we considered only 2 ICD-9 codes for influenza. Second, it is not known whether our findings extend beyond our region. Last, changes in health-seeking behavior in 2009–10 versus 2013–14 were not addressed.

## Conclusions

We hypothesize that the emergence of an influenza virus variant bearing the D225G polymorphism enabled the 2013 H1N1 virus to infect lower and upper respiratory tract cells, thereby contributing to the increased severity of the 2013–14 influenza season in our region. Our findings highlight the importance of monitoring genetic changes in the 2013 H1N1 virus to predict the effect of future influenza viruses.
